# Pyroglutamate-modified Aβ(3-42) affects aggregation kinetics of Aβ(1-42) by accelerating primary and secondary pathways†
†Electronic supplementary information (ESI) available. See DOI: 10.1039/c6sc04797a



**DOI:** 10.1039/c6sc04797a

**Published:** 2017-05-05

**Authors:** C. Dammers, M. Schwarten, A. K. Buell, D. Willbold

**Affiliations:** a Institute of Complex Systems (ICS-6) Structural Biochemistry , Forschungszentrum Jülich , 52425 Jülich , Germany . Email: d.willbold@fz-juelich.de; b Institut für Physikalische Biologie , Heinrich-Heine-Universität Düsseldorf , 40225 Düsseldorf , Germany

## Abstract

The aggregation into amyloid fibrils of amyloid-β (Aβ) peptides is a hallmark of Alzheimer's disease. A variety of Aβ peptides have been discovered *in vivo*, with pyroglutamate-modified Aβ (pEAβ) forming a significant proportion. pEAβ is mainly localized in the core of plaques, suggesting a possible role in inducing and facilitating Aβ oligomerization and accumulation. Despite this potential importance, the aggregation mechanism of pEAβ and its influence on the aggregation kinetics of other Aβ variants have not yet been elucidated. Here we show that pEAβ(3-42) forms fibrils much faster than Aβ(1-42) and the critical concentration above which aggregation was observed was drastically decreased by one order of magnitude compared to Aβ(1-42). We elucidated the co-aggregation mechanism of Aβ(1-42) with pEAβ(3-42). At concentrations at which both species do not aggregate as homofibrils, mixtures of pEAβ(3-42) and Aβ(1-42) aggregate, suggesting the formation of mixed nuclei. We show that the presence of pEAβ(3-42) monomers increases the rate of primary nucleation of Aβ(1-42) and that fibrils of pEAβ(3-42) serve as highly efficient templates for elongation and catalytic surfaces for secondary nucleation of Aβ(1-42). On the other hand, the addition of Aβ(1-42) monomers drastically decelerates the primary and secondary nucleation of pEAβ(3-42) while not altering the pEAβ(3-42) elongation rate. In addition, even moderate concentrations of fibrillar Aβ(1-42) prevent pEAβ(3-42) aggregation, likely due to non-reactive binding of pEAβ(3-42) monomers to the surfaces of Aβ(1-42) fibrils. Thus, pEAβ(3-42) accelerates aggregation of Aβ(1-42) by affecting all individual reaction steps of the aggregation process while Aβ(1-42) dramatically slows down the primary and secondary nucleation of pEAβ(3-42).

## Abstract 2

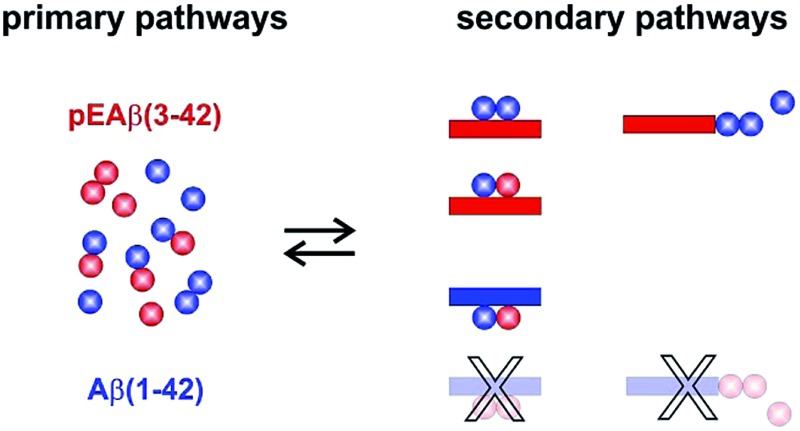
Fibrillary and monomeric pyroglutamate-modified amyloid-β(3-42) accelerates all individual reaction steps of full-length amyloid-β(1-42) and act as a subsequent seeding species.

## Introduction

Extracellular insoluble fibrillar structures are a pathological hallmark of Alzheimer's disease (AD) and are mainly composed of depositions of amyloid-β peptides (Aβ).^1,2^ Several Aβ variants have been found in *in vivo* deposits, with N-terminally truncated Aβ variants as a significant proportion.^3,4^ Pyroglutamate-modified Aβ (pEAβ) variants, especially pEAβ(3-42), have been demonstrated to be the predominant isoforms amongst these.^5–7^

Modification to pEAβ results in altered biophysical and biochemical characteristics with potentially severe pathological consequences. Formation of the intramolecular lactam ring increases its resistance to degradation by amino peptidases and therefore the overall stability.^8^ Since the modification to pEAβ(3-*x*) results in the loss of one positive and two negative charges, the enhanced hydrophobicity and decreased electrostatic repulsion of pEAβ(3-*x*) leads to dramatically accelerated aggregation kinetics compared to Aβ(1-*x*) – independently of the C-terminal length.^9^ Structural analysis on pEAβ(3-40/42) and Aβ(1-40/42) isoforms using solution state nuclear magnetic resonance (NMR) spectroscopy indicate that the pyroglutamate-modified variants have an increased tendency to form β-sheet rich structures compared to their non-truncated isoforms.^10,11^

Levels of pEAβ ending with position 42 were found to be always higher than C-terminally shortened species;^4,12^ pEAβ(3-42) represents 25% of the total Aβ in senile plaques.^4^ Moreover, pEAβ(3-*x*) was detected in the core of amyloid aggregates *in vivo*, leading to the hypothesis that pEAβ deposition plays a central role in initiating the aggregation of full-length Aβ.^7,13–15^ In general, pEAβ was shown to be more likely to form β-sheet structures and has an enhanced aggregation propensity compared to the not N-terminally truncated peptide underlining its potential role in seeding Aβ oligomerization and accumulation.^9,16–18^ The N-terminus plays an important role in determining the thermodynamic stability of the fibrils^19^ and the pEAβ(3-*x*) variants have been proposed to lead to enhanced fragmentation.^20^ The increased aggregation propensity is in line with the hypothesis that pEAβ(3-42) fibrils act as a template for full-length Aβ species.

As a major species in diffuse and compacted plaques in the human AD brain, pEAβ(3-42) is consequently an emerging target for active as well as passive immunotherapy trials.^21^ Non-truncated Aβ(1-40/42) are physiologically generated as products of normal APP turnover^22,23^ whereas the occurrence of a significant proportion of pEAβ(3-40/42) is the result of a side reaction of the enzyme glutaminyl cyclase (QC) and alters the amyloidogenicity and toxicity of the Aβ molecule.^21^ Thus, new immunotherapy strategies focus on Aβ peptides with high toxic potential such as pEAβ(3-40/42).^24^ Although the detailed aggregation mechanism and the influence of pEAβ(3-42) on the kinetics of other Aβ variants are still unknown, the aggregation mechanism of the unmodified Aβ(1-42) peptide has been analysed in detail. Using a combination of kinetic experiments and theoretical analysis, it was demonstrated that Aβ(1-42) aggregation is dominated by autocatalytic secondary nucleation under quiescent conditions.^25–27^ Furthermore, it was shown that Aβ(1-42) and its C-terminally truncated version Aβ(1-40) do not significantly co-aggregate.^28^ Interestingly, the insertion of N-terminal extensions of Aβ(1-42) allows cross-seeding and co-aggregation^29^ indicating that the presence of N-terminally modified forms of the peptide can have a strong effect on the aggregation kinetics of the full-length sequence.

In the present study, we have elucidated the co-aggregation mechanism of Aβ(1-42) with the more toxic and aggregation prone variant pEAβ(3-42) by kinetic studies using highly pure recombinant peptides.^30^

## Results and discussion

### pEAβ(3-42) aggregation kinetics indicate increased secondary pathways

**Fig. 1:**
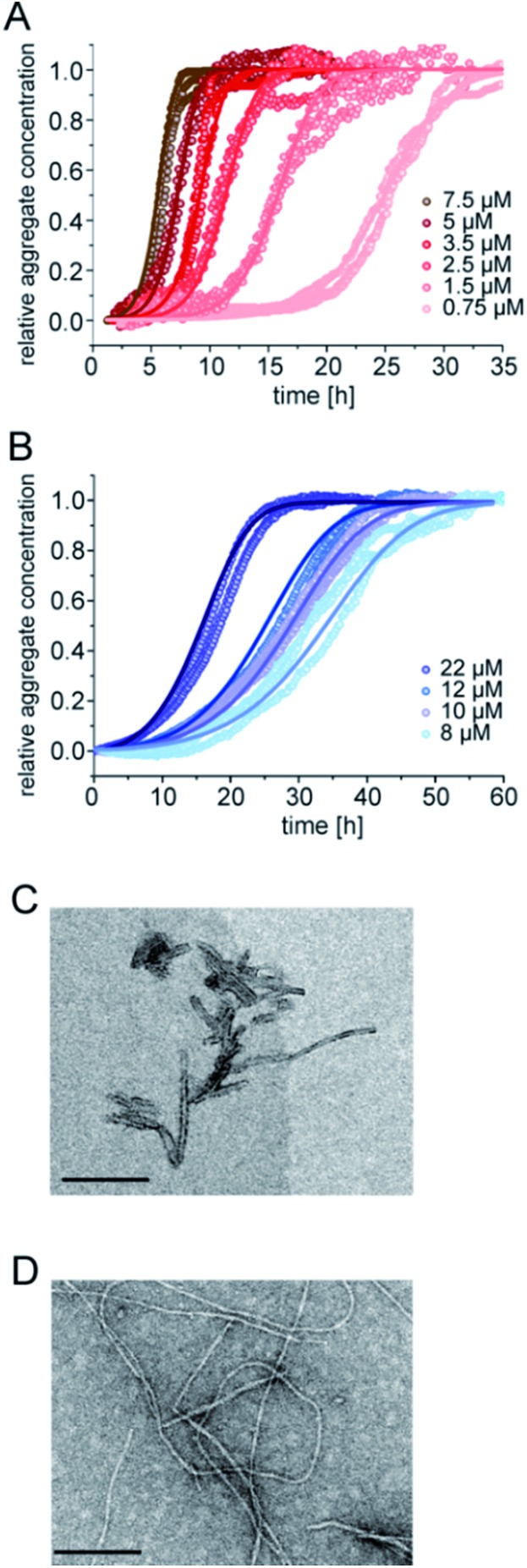
Aggregation kinetics of Aβ(1-42) and pEAβ(3-42) and TEM images of fibrils. Aggregation kinetics of pEAβ(3-42) (A) and Aβ(1-42) (B) monitored by ThT fluorescence. Analysis was performed using the software AmyloFit^31^ after normalizing the raw data according to their initial peptide monomer concentration. TEM images of pEAβ(3-42) (C) and Aβ(1-42) (D) were obtained after seven days of incubation (the black scale bar corresponds to 200 nm).

The aggregation kinetics of pure samples of pEAβ(3-42) and Aβ(1-42) were monitored by Thioflavin-T (ThT) fluorescence. Theoretical analysis of aggregation kinetics was performed using the software AmyloFit.^31^ First, we performed kinetic experiments of both Aβ(1-42) and pEAβ(3-42) at various concentrations and found that pEAβ(3-42) aggregates much faster than Aβ(1-42). Furthermore, the concentration above which aggregation was observed within the timescale of our experiments was decreased by one order of magnitude for pEAβ(3-42) compared to Aβ(1-42) (Fig. 1A and B). While Aβ(1-42) assembles into networks of long thin fibrils, pEAβ(3-42) fibrils are on average much shorter (Fig. 1C and D). This could be either caused by a higher fragmentation rate, as proposed recently,^20^ or alternatively by a higher nucleation rate, with both scenarios leading to the formation of more, but shorter aggregates from a given initial concentration of monomeric peptide.^32^

The scaling exponents from a log–log plot of half time against initial peptide concentration are in both cases close to –0.5 (–0.63 for pEAβ(3-42) and –0.76 for Aβ(1-42)) (Fig. S1†), which is indicative of either a fragmentation dominated mechanism,^33^ or of a saturated secondary nucleation mechanism.^34^ It has previously been shown that the surface-catalysed secondary nucleation mechanism of the Aβ(1-40) peptide becomes with increasing initial peptide concentration successively less concentration dependent as the binding sites on the surface of the fibrils become saturated at concentrations above *ca.* 10 μM.^34^ Our results are consistent with this picture, given that we are exploring a concentration range of 8–22 μM in the case of the Aβ(1-42) peptide. The finding that the scaling exponent of pEAβ(3-42) is lower than that for Aβ(1-42) peptide, despite the fact that the experiments with the former were performed at lower concentrations, suggests a very high affinity of the pEAβ(3-42) peptide for its fibril surfaces, leading to saturation of the binding sites on the fibril surfaces already at lower concentrations, and hence to a weaker concentration dependence.

### pEAβ(3-42) and Aβ(1-42) monomers co-aggregate

**Fig. 2:**
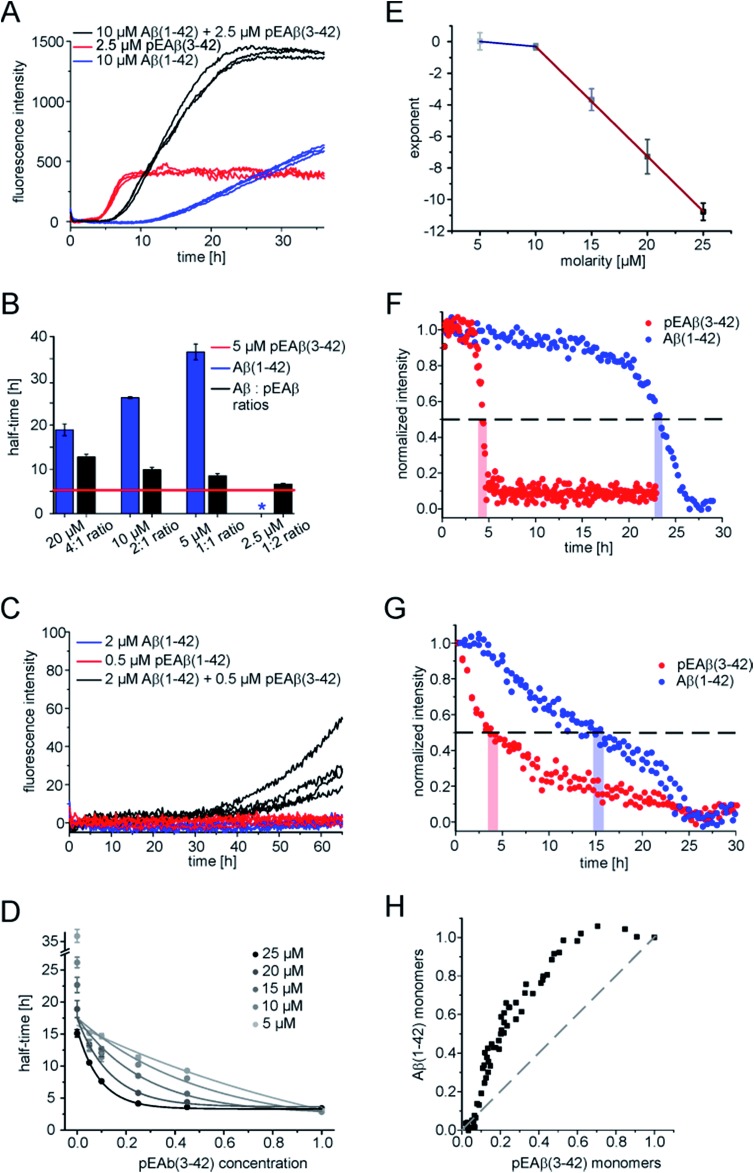
Interaction of pEAβ(3-42) and Aβ(1-42) monomers. (A) Aggregation kinetics of Aβ(1-42), pEAβ(3-42) and mixtures of both peptides measured by ThT assay. (B) Half-times of Aβ(1-42), pEAβ(3-42) and mixtures of both determined from normalized aggregation kinetic data. The asterisk indicates that no aggregation could be detected. (C) Aggregation monitored by ThT assay of pEAβ(3-42) and Aβ(1-42) below their critical concentration and mixtures of both. (D) Half-times of different concentrations of pEAβ(3-42) and Aβ(1-42) mixtures plotted as a function of the total pEAβ percentage and fitted with an exponential function. (E) Coefficients of a fit of an exponential function to the decrease in half-time of pEAβ(3-42) and Aβ(1-42) monomer mixtures plotted as a function of the total molarity. The solid lines are linear fits (red: slope –0.69 ± 0.01, blue –0.07 ± 0.03) (F) normalized signal intensity gained from NMR spectroscopy time series of 10 μM homomolecular pEAβ(3-42) or Aβ(1-42). The dashed line marks 50% signal intensity and the red and blue bars indicate the times when half of the monomer of pEAβ(3-42) or Aβ(1-42) was consumed. (G) Normalized intensity gained from NMR spectra monitoring monomer depletion of either 10 μM pEAβ(3-42) or Aβ(1-42), respectively, starting from equimolar mixtures and the percentage of Aβ(1-42) monomers plotted against pEAβ(3-42) monomers (H).

In order to obtain further insight into the origin of the faster aggregation of pEAβ(3-42) compared to Aβ(1-42), the aggregation kinetics of pure peptide were compared with those of mixtures of varying composition and total concentration (Fig. 2A and B and S2†).

Heteromolecular mixtures of monomeric pEAβ(3-42) and Aβ(1-42) displayed a single sigmoidal aggregation kinetic curve, indicating that both species undergo co-aggregation. The half-times, which are determined as the times when half of the final aggregate mass was reached, were calculated and compared with the kinetics of homomolecular aggregation of pEAβ(3-42) or Aβ(1-42), respectively (Fig. 2B). The half-times of the mixtures increase significantly with increasing Aβ(1-42) concentration, compared to the aggregation of pure pEAβ(3-42), even when Aβ(1-42) is added at a concentration where it does not display aggregation on its own.

In addition, amyloid fibril formation is also observed in mixtures of both species at concentrations of Aβ(1-42) (2 μM) and pEAβ(3-42) (0.5 μM) where each of the species individually does not show any aggregation under the same conditions (Fig. 2C). This finding further supports the hypothesis that the two species interact already at the level of the primary nucleation step.

The aggregation kinetics of different monomeric mixtures of Aβ(1-42) and pEAβ(3-42), varying from 0% to 100% of pEAβ(3-42) at total concentrations from 5 to 25 μM were analysed. The data were normalised, the half-times were calculated and plotted as a function of the proportion of pEAβ(3-42) concentration for each total concentration set that had been measured (Fig. 2D). In order to be able to quantify the differences between the individual data sets, we fitted the decrease in half-time as a function of the proportion of pEAβ(3-42) with a single exponential function. We would like to stress that this does not imply that the underlying functional behaviour is necessarily exponential, but an exponential function yielded the best fit results. The exponents from the fits (numerical values are shown in Table S1†) were then plotted as a function of the total concentration from 10 to 25 μM (Fig. 2E). The results show, that the higher the total peptide concentration, the smaller the percentage of pEAβ(3-42) needed to significantly decrease the aggregation half-times, suggesting that the absolute concentration of pEAβ(3-42) is important for the accelerating effect. Two approximately linear regimes were observed, intersecting at a total concentration around 10 μM, which was shown to be the concentration where the surface-dependent secondary nucleation of Aβ(1-40) becomes saturated and hence concentration independent.^34^ This change in behaviour suggests that the mechanism of co-aggregation of both species varies as a function of the total Aβ concentration. The finding that the accelerating effect of pEAβ(3-42) is more pronounced at higher concentrations (above 10 μM), where secondary nucleation is likely to be concentration independent due to saturation (see above) suggests that the predominant process responsible for the acceleration at these higher concentrations is primary nucleation. The rate of primary nucleation cannot be saturated and hence does not show a concentration-independent regime.

### Monomer depletion of pEAβ(3-42) and Aβ(1-42) monitored during co-nucleation

As ThT assays are almost exclusively sensitive to the formation of fibrillar species, we decided to also monitor the evolution of the concentration of monomeric Aβ *via* nuclear magnetic resonance (NMR) spectroscopy, based on the invisibility in conventional NMR experiments of large aggregated species.^35^ The monomer depletion of either Aβ(1-42) or pEAβ(3-42) as homomolecular samples are complementary to the ThT aggregation kinetics (Fig. 2F). An experiment with a starting concentration of 10 μM Aβ(1-42) shows that half the peptide has become insoluble after 23 h and reaches a minimum in signal intensity at 27.5 h. In contrast, soluble pEAβ(3-42) monomers have decreased to 50% of the initial concentration after 4 h and the signal has almost completely disappeared after 5 h. Interestingly, in equimolar mixtures with the same concentrations of pEAβ(3-42) and Aβ(1-42), the time courses of monomer depletion of both species differ significantly from those obtained from experiments with pure peptides (Fig. 2G). In this mixture, the loss of soluble Aβ(1-42) is faster than in a pure sample of Aβ(1-42), whereas the loss of soluble pEAβ(3-42) is slower than in the pure case, providing additional support for the hypothesis that both species interact with each other throughout the time course of aggregation. The concentration of monomeric pEAβ(3-42) decreases faster than that of Aβ(1-42) monomers (Fig. 2F and G). Although pEAβ(3-42) and Aβ(1-42) show different half-times, both NMR signal intensities are minimal after 25 h, indicating the formation of mixed aggregates up to the end of the aggregation reaction. Plotting the Aβ(1-42) monomer concentration as a function of the pEAβ(3-42) monomer concentration illustrates that the Aβ(1-42) monomer concentration stays close to its initial value until the pEAβ(3-42) concentration has decreased to approximately 50% (Fig. 2H). Thus, the aggregation of Aβ(1-42) is accelerated most strongly in the presence of pEAβ(3-42) fibrils indicating that fibrils of pEAβ(3-42) provide efficient nucleation sites for Aβ(1-42) monomers.

### pEAβ(3-42) fibrils as a highly catalytic surface for secondary pathways

**Fig. 3:**
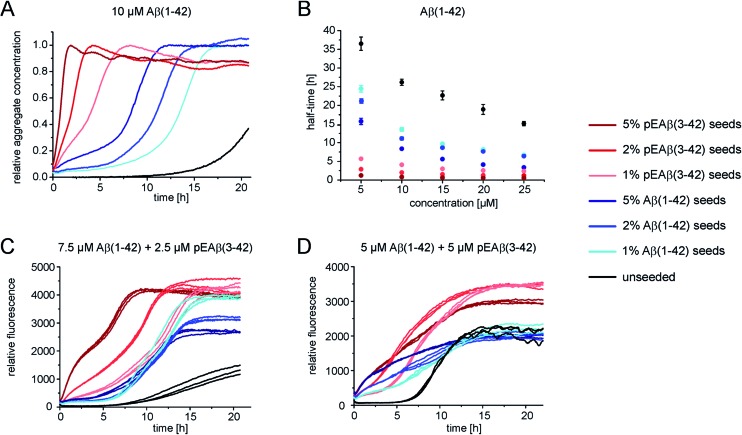
Seeded aggregation kinetics of Aβ(1-42) monomers. (A) Normalized aggregation kinetics of 10 μM Aβ(1-42) monomers, unseeded and seeded with different concentrations of Aβ(1-42) or pEAβ(3-42) fibrils and normalized according to the aggregate concentration. (B) Half-times of varying Aβ(1-42) monomer concentrations seeded with different amounts of fibrils. (C) Aggregation kinetics of 7.5 μM Aβ(1-42) and 2.5 μM pEAβ(3-42) and of equimolar 5 μM Aβ(1-42) and pEAβ(3-42) (D) seeded with Aβ(1-42) and pEAβ(3-42) fibrils in varying concentrations.

In order to gain further insight into the role of aggregates in the mechanism of co-aggregation, we performed kinetic assays with preformed seed fibrils that can act as templates for elongation and as catalytic surfaces for secondary nucleation. Experiments with Aβ(1-42) monomers showed that pEAβ(3-42) fibrils are very efficient seeds for these monomers (Fig. 3A and S3†). At high pEAβ(3-42) seed concentration (5%) Aβ(1-42) aggregation is dominated by elongation, as evidenced by the concave aggregation time course, whereas the aggregation curves at lower seed concentrations show the typical convex shape indicative of an accelerating aggregation reaction and hence of the contribution of secondary processes.^36^ Compared to the seeding effect of Aβ(1-42) fibrils, only small amounts of pEAβ(3-42) fibrils are needed to accelerate Aβ(1-42) aggregation drastically, and this accelerating effect is only weakly dependent on the total initial concentration of monomeric Aβ(1-42) as shown by analysis of the half-times (Fig. 3B). Without a complete kinetic analysis of the co-aggregating system, it is difficult to quantify the relative contributions of elongation and secondary nucleation to the highly efficient seeding by pEAβ(3-42) fibrils. A higher elongation rate could for example be caused simply by the larger number of growth-competent ends per unit mass of fibrils, as the pEAβ(3-42) fibrils are on average shorter (Fig. 1C). Furthermore, differences in elongation or indeed secondary nucleation rate could also be caused by differences in the molecular structure of the fibrils, both at the ends and on the surface. The experimental finding of a decreasing concentration dependence of the accelerating effect with increasing initial monomer concentration suggests that a potential contribution from secondary nucleation is saturating.

Interesting behaviour is observed when mixtures of monomeric Aβ(1-42) and pEAβ(3-42) are seeded. Some of the kinetic curves are first concave, then convex and then concave again (Fig. 3C and D), in particular those reactions that were seeded with pEAβ(3-42). This resembles the unseeded aggregation curves that have been reported for mixtures of the Aβ(1-42) and Aβ(1-40) peptides,^28^ which were shown not to co-aggregate significantly. However, the results of our unseeded kinetic experiments by ThT and NMR of mixtures of Aβ(1-42) and pEAβ(3-42) (Fig. 2C and G and black curves in Fig. 3A, C and D) do not show biphasic behaviour and present clear evidence for co-aggregation. The biphasic aggregation curves in the presence of seeds can therefore be explained through a preferential incorporation of one type of soluble Aβ into the seed aggregates, followed by the slower and less efficient incorporation of the remaining monomer type. The later acceleration in aggregation rate is then likely to be due to secondary nucleation of the remaining soluble Aβ on the surface of the mixed fibrils. This phenomenon is observed in monomer mixtures containing a molar excess of Aβ(1-42), *e.g.* 7.5 μM Aβ(1-42) and 2.5 μM pEAβ(3-42) monomers (Fig. 3C).

The two-phase kinetic behaviour is less pronounced in equimolar mixtures, when the total amount of pEAβ(3-42) monomers is increased (Fig. 3D). At equimolar ratio the direct interaction of pEAβ(3-42) monomers with Aβ(1-42) monomers is likely to play a more pronounced role. This interaction, which leads to the formation of mixed primary nuclei, appears to weaken the difference between the effects of the two types of fibrils, as the initial slopes are very similar until secondary nucleation becomes manifest through an increase in the aggregation rate. Both types of seeds lead to very similar initial aggregation rates under those conditions, that only depend on the total fibril mass, but the reactions that were seeded with pEAβ(3-42) fibrils subsequently show a clear increase in rate, indicative of rapid secondary nucleation. This finding suggests that the structural features of the pEAβ(3-42) fibrils render their surfaces more amenable to secondary nucleation.

Overall, this data is consistent with an incorporation of Aβ(1-42) into pEAβ(3-42) seeds that is more efficient than the incorporation of pEAβ(3-42), *i.e.* heteromolecular elongation is more efficient than homomolecular elongation. This unexpected behaviour is for example suggested by the more rapid aggregation in Fig. 3A compared to Fig. 3C (red curves). It has to be noted, however, that seeded aggregation experiments need to be compared with care, as the aggregation rate depends on the concentration of growth-competent seeds, which is difficult to control and determine precisely, as it is a function of the exact length distribution of the seeds.

In order to obtain insight into the structural consequences of the complex interaction behaviour described above, we have acquired atomic force microscopy (AFM) and transmission electron microscopy (TEM) images of mixtures of 5 μM pEAβ(3-42) and 5 μM Aβ(1-42) that were either unseeded, seeded with 5% pEAβ(3-42) fibrils or with 5% Aβ(1-42) fibrils (Fig. S4†). These images show that in each case, long fibrils and smaller species coexist.

Elucidation of the full mechanism of coaggregation as a function of seed type and concentration, as well as monomer ratio and concentration, requires a systematic and comprehensive imaging study, combined with a determination of the ratio of soluble peptides as a function of time for each of these conditions, which will be the subject of a future study.

### Aβ(1-42) fibrils are not suitable for cross-seeding of pEAβ(3-42)

**Fig. 4:**
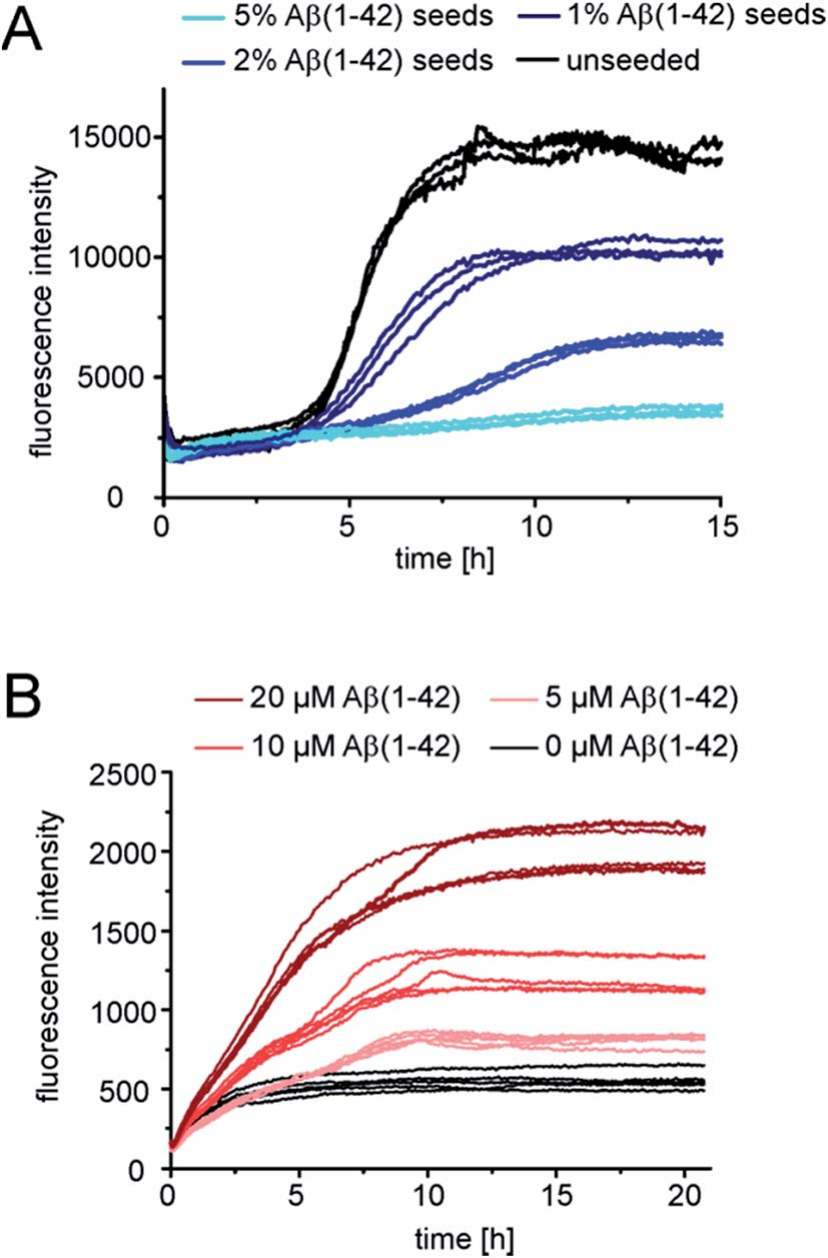
Seeded kinetic assays of pEAβ(3-42) monomers. (A) Aggregation kinetics of 2.5 μM pEAβ(3-42) unseeded and seeded with varying concentrations of Aβ(1-42) fibrils. (B) Aggregation kinetics of 5 μM pEAβ(3-42) seeded with 20% pEAβ(3-42) fibrils and additional Aβ(1-42) monomers in varying concentrations.

Next we tested whether Aβ(1-42) fibrils are able to seed pure monomeric pEAβ(3-42). Aggregation kinetics of pEAβ(3-42) monomers was delayed even in the presence of 1% Aβ(1-42) fibrils and the total aggregate mass (as deduced from fluorescence intensity) was decreased (Fig. 4A) compared to the unseeded case. By adding 5% Aβ(1-42) fibrils the fibrillation of pEAβ(3-42) was almost completely inhibited, as judged by ThT fluorescence. This phenomenon is dependent on the Aβ(1-42) fibril concentration and was also observed for equimolar mixtures of both monomeric species. Aggregation is inhibited and decreased if the amount of Aβ(1-42) fibrils is large enough, *i.e.* 20% (Fig. S5†). This result suggests that pEAβ(3-42) monomers can attach to the Aβ(1-42) fibril surface in a way that does not allow secondary nucleation. In order to further support this hypothesis, we performed NMR experiments, whereby we measured the decrease in NMR signal of soluble pEAβ(3-42) upon addition of 5% Aβ(1-42) (Fig. S6†). Within the time scale of the NMR experiment (*ca.* 10 min), we observe a very significant (*ca.* 50%) decrease in signal intensity, suggesting that half of the soluble pEAβ(3-42) peptide strongly interacts with the Aβ(1-42) fibrils, with a very high stoichiometry of the order of 10 : 1. We have acquired AFM images of a mixture of pEAβ(3-42) and 5% Aβ(1-42) fibrils (Fig. S7†) that show clusters of fibrils that appear to be coated in monomer. Thus, we conclude that pEAβ(3-42) binds with a high affinity, as well as stoichiometry, to Aβ(1-42) fibrils, and this interaction does not lead to self-replication through secondary nucleation.

Non-reactive surface binding and thus the absence of self-replication of amyloidogenic peptides was recently postulated, based on the results of coarse-grained simulations.^37^ The overall reaction rate of secondary nucleation was shown to be determined by the protein adsorption and subsequent oligomer formation on the fibril surface, the latter being only possible at intermediate protein-fibril interaction strengths. Too strong interactions render the surface-bound peptides inert.

Although Aβ(1-42) monomers drastically decelerate pEAβ(3-42) primary and secondary nucleation, the pEAβ(3-42) elongation rate, as evaluated from the initial slope of the aggregation curve, is not significantly affected as demonstrated in the presence of high amounts of pEAβ(3-42) fibrils (Fig. 4B and S8†). This suggests that the rates of incorporation of both Aβ(1-42) and pEAβ(3-42) into pEAβ(3-42) fibrils are not substantially different and that therefore the latter provide an efficient template for elongation of both types of peptide, whereas Aβ(1-42) fibrils can only act as homomolecular templates.

## Conclusions

**Fig. 5:**
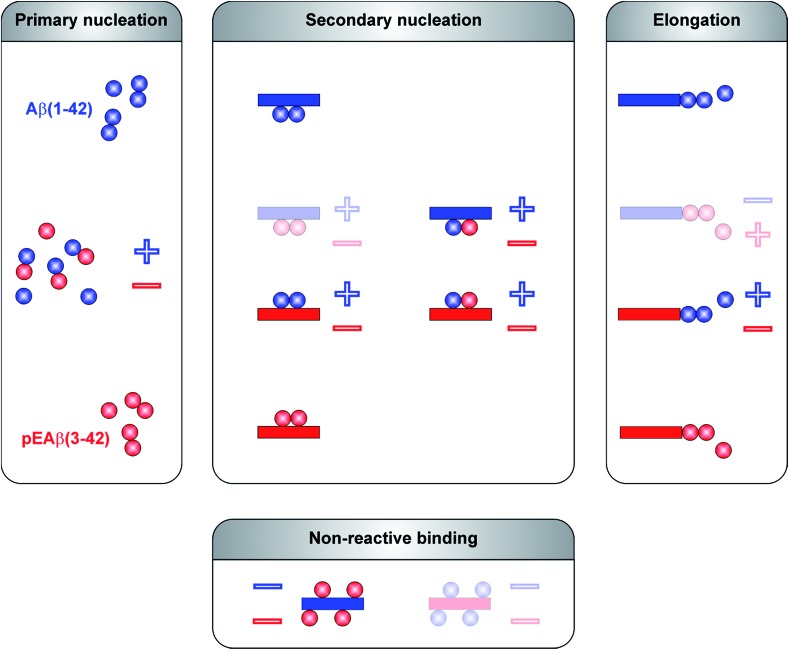
Reaction scheme of molecular processes during Aβ oligomerization and aggregation. Microscopic events of Aβ(1-42) are displayed in blue and in red for pEAβ(3-42). Interactions are separated between primary pathways and secondary pathways as well as non-reactive binding. Individual steps which are accelerated in the presence of the other Aβ species are marked with a plus and with a minus in case of a decelerating effect. Molecular events which can be excluded based on our data are shown as faded schemes.

The aggregation mechanism of Aβ(1-42) was previously shown to be secondary nucleation dominated under quiescent conditions.^25–27^ In contrast to C-terminally truncated Aβ species which aggregate into homofibrils without co-nucleation,^28^ N-terminal modifications are able to co-aggregate.^29^ Here, we have elucidated the mechanism of co-aggregation of Aβ(1-42) with the more toxic and the more aggregation prone variant pEAβ(3-42) and our results are summarized schematically in Fig. 5. The presence of small amounts of pEAβ(3-42) monomers increases the primary nucleation rate of Aβ(1-42) and pEAβ(3-42) fibrils serve as highly efficient seeds for both elongation and (auto)catalytic secondary nucleation of non-N-terminally truncated Aβ monomers while pEAβ(3-42) aggregation itself is slowed down through the presence of Aβ(1-42) monomers. In addition, Aβ(1-42) fibrils are not suitable as templates for the incorporation of monomeric pEAβ(3-42) but can even prevent pEAβ(3-42) aggregation at high fibril concentrations, presumably due to the non-reactive binding of pEAβ(3-42) monomers to Aβ(1-42) fibril surfaces. Thus, pEAβ(3-42) catalyses aggregation of Aβ(1-42) affecting all reaction processes while Aβ(1-42) dramatically slows down pEAβ(3-42) primary and secondary pathways by non-reactive surface binding. Therefore, the presence of even relatively small amounts of additional isoforms can very significantly change the aggregation behavior of the Aβ(1-42) peptide. The insight gained in this study will enable a more detailed understanding of the aggregation dynamics *in vivo*, where complex mixtures of various isoforms of the Aβ peptide are likely to be present.

## Methods

### Recombinant Aβ peptides

Expression and purification of pEAβ(3-42) was performed as described recently.^30^ Briefly, Aβ(E3Q-42) was expressed in *E. coli* BL21 (DE3) pLysS as a fusion protein and purified *via* immobilized metal affinity chromatography (IMAC) and reversed-phase high performance liquid chromatography (RP-HPLC). The fusion-tag was cleaved by tobacco etch virus (TEV) protease and RP-HPLC purified Aβ(E3Q-42) was non-enzymatically converted to pEAβ(3-42) under mild acidic conditions. Final pEAβ(3-42) was obtained in purities of ≥98%. Recombinant Aβ(1-42) was purchased from Isoloid (Düsseldorf, Germany).

### Sample preparation

Samples were prepared in Protein LowBinding tubes (Eppendorf AG, 230 Hamburg, Germany). The purified Aβ peptides were dissolved in 1,1,1,3,3,3-hexafluoro-2-propanol (HFIP, Sigma-Aldrich, Hannover, Germany), incubated for 3 days at room temperature for disaggregation. Monomerised peptides were lyophilized directly from HFIP and stored at room temperature. Prior to use, purified peptides were again dissolved in HFIP, monomerised overnight and final sample aliquots were prepared and lyophilized.

### Preparation of fibrils

Lyophilized pEAβ(3-42) and Aβ(1-42) were dissolved in 10 mM sodium phosphate buffer pH 7.4 to a final protein concentration of 50 μM, respectively, and incubated at 37 °C for seven days without agitation.

### Kinetic assays of monomers

Aβ(1-42) and pEAβ(3-42) dissolved in HFIP were aliquoted, lyophilized and directly dissolved in 10 mM ice-cold sodium phosphate buffer pH 7.4 including 10 μm ThT. The solutions were vortexed; dilutions series were prepared and gently mixed by pipetting up and down. The final concentration of prepared samples was ranging between 0.5 μM and 25 μM, depending on the peptide. Aggregation assays were performed in black non-binding 96-well plates (Sigma-Aldrich, Germany) with 100 μL per well as triplicates at 37 °C in quiescent conditions. Fluorescence was monitored using a microplate reader (PolarStar Optima, BMG, Offenburg, Germany) with 440 and 492 nm excitation and emission filters, respectively, in bottom-read mode.

### Kinetic assays of monomer mixtures

Monomer mixtures of Aβ(1-42) and pEAβ(3-42) were prepared with stock solutions dissolved in HFIP, respectively, mixed in reaction tubes and lyophilized prior to use. Mixed monomers were dissolved in 10 mM ice-cold sodium phosphate buffer pH 7.4 including 10 μm ThT, vortexed and sonicated for 2 min and dilutions series were prepared. The final concentrations of the mixtures were in a range of 0.5 to 25 μM. Aggregation assays were performed in triplicates as described above.

### Seeded kinetic assays

Samples for seeded kinetic assays of Aβ(1-42) and pEAβ(3-42) monomer homogenates as well as mixtures were prepared as described above and dissolved in ice-cold 10 mM sodium phosphate pH 7.4 and 10 μM ThT. Fibrils were sonicated for 2 min prior to use and added to the reaction tube in final concentrations of 1, 2 or 5% in monomer equivalents. Dilution series were prepared and concentrations of final samples were ranging from 0.5 μM to 25 μM with 1, 2 or 5% seeds. Aggregation assays were performed in triplicates as described above.

### Analysis of aggregation kinetics

Analysis of homomolecular Aβ(1-42) and pEAβ(3-42) aggregation kinetics was performed using the online tool AmyloFit published by Meisl, Knowles and coworkers.^31^ Briefly, kinetic datasets were normalized to their final aggregate concentration, plotted as a function of time and fitted using the appropriate model.^32,33^ Heteromolecular data could not be analysed *via* this tool as multiple processes occurred during aggregation. This data was analysed either by plotting raw or normalized data and especially halftimes; the point where aggregation has reached half its maximum.

### TEM

Fibrils were absorbed on formvar/carbon coated copper grids (S162, Plano, Wetzlar, Germany) for 5 min and washed with water. Negative staining was performed by incubation with 2% (w/v) uranylacetate for 45 s. Images were taken with a Libra 120 transmission electron microscope (Zeiss, Oberkochen, Germany) at 120 kV.

### NMR spectroscopy

Lyophilized natural abundant pEAβ(3-42) and [U–^13^C, ^15^N]-Aβ(1-42) as well as equimolar mixtures were dissolved in 10 mM sodium phosphate buffer pH 7.4 including 5% D_2_O to a final protein concentration of 10 μM of each Aβ species. Titration experiments were performed by dissolving pEAβ(3-42) to a final concentration of 5 μM. Aβ(1-42) fibrils were then directly added. NMR spectra were acquired using a Varian 800 MHz or a Bruker 700 MHz spectrometer equipped with cryogenically cooled z-pulse-field-gradient probes at 37 °C. Methyl-proton signals were obtained from CN-filtered-noesy,^38^ gChsqc^39^ or zgpr pulse sequences, respectively. Spectra were processed with NMRPipe^40^ and evaluated with CCPNmr Analysis.^41^
