# Supplementary material for: Pyroglutamate-modified Aβ(3-42) affects aggregation kinetics of Aβ(1-42) by accelerating primary and secondary pathways
Source: Chem Sci. 2017 May 5;8(7):4996–5004. doi: 10.1039/c6sc04797a (PMC5612032; doi:10.1039/c6sc04797a)
Supplement: Supplementary file 1 [file SC-008-C6SC04797A-s001.pdf]

Electronic Supplementary Material (ESI) for Chemical Science.
This journal is © The Royal Society of Chemistry 2017

Pyroglutamate-modified Aβ(3-42) Affects Aggregation Kinetics of Aβ(1-42) by Accelerating Primary and Secondary Pathways

C. Dammers,[a] M. Schwarten,[a] A. K. Buell[b] and D. Willbold[a,b]

[a] Institute of Complex Systems (ICS-6) Structural Biochemistry, Forschungszentrum Jülich, 52425 Jülich, Germany
[b] Institut für Physikalische Biologie, Heinrich-Heine-Universität Düsseldorf, 40225 Düsseldorf, Germany.

Supporting Information

Table S1. Exponential coefficient of the fitted functions representing the pEAβ(3-42) concentration-dependent decrease in half-time of monomer mixtures varying from 0 % to 100 % pEAβ(3-42) in total peptide concentrations from 5 to 25 μM.

Total molarity	Exponential coefficient	Standard error
25 μM	-10.77	0.54
20 μM	-7.28	1.09
15 μM	-3.67	0.69
10 μM	-0.39	0.38
5 μM	-0.02	0.54

Supplementary Figures

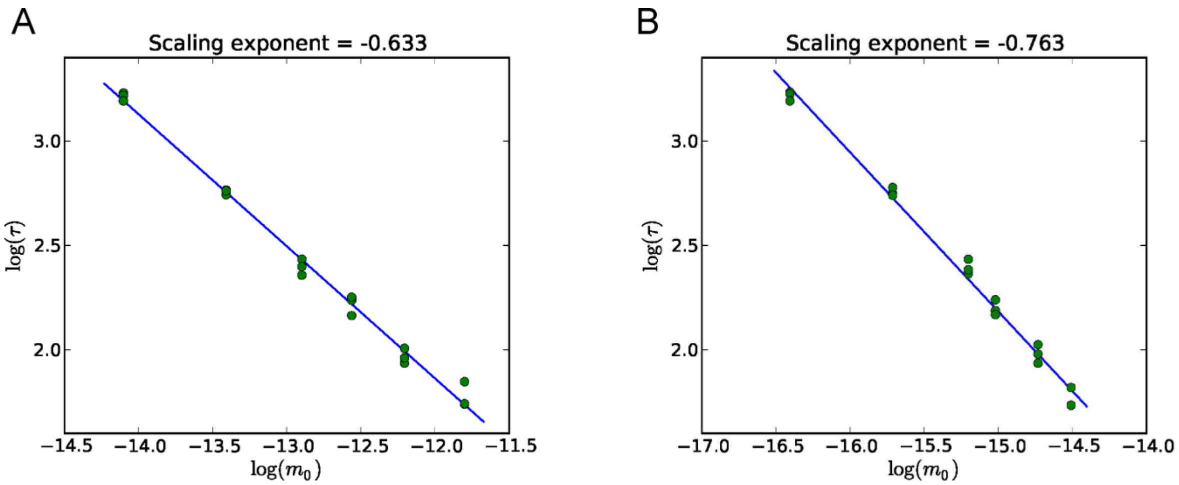

Figure S1. Log-log plot of the half-times of pEAβ(3-42) (A) and Aβ(1-42) (B) against initial peptide concentration. The half-times were calculated and plotted using the software AmyloFit (1).

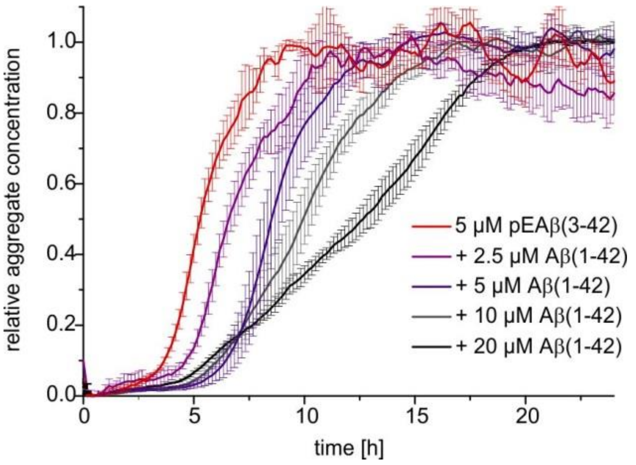

Figure S2. Aggregation kinetics of Aβ(1-42) and pEAβ(3-42) mixtures. 5 μM pEAβ(3-42) monomers were mixed with different Aβ(1-42) concentrations and aggregation was monitored by ThT assay. Raw data of triplicates was averaged (see error bars) and normalized to the relative aggregate concentration.

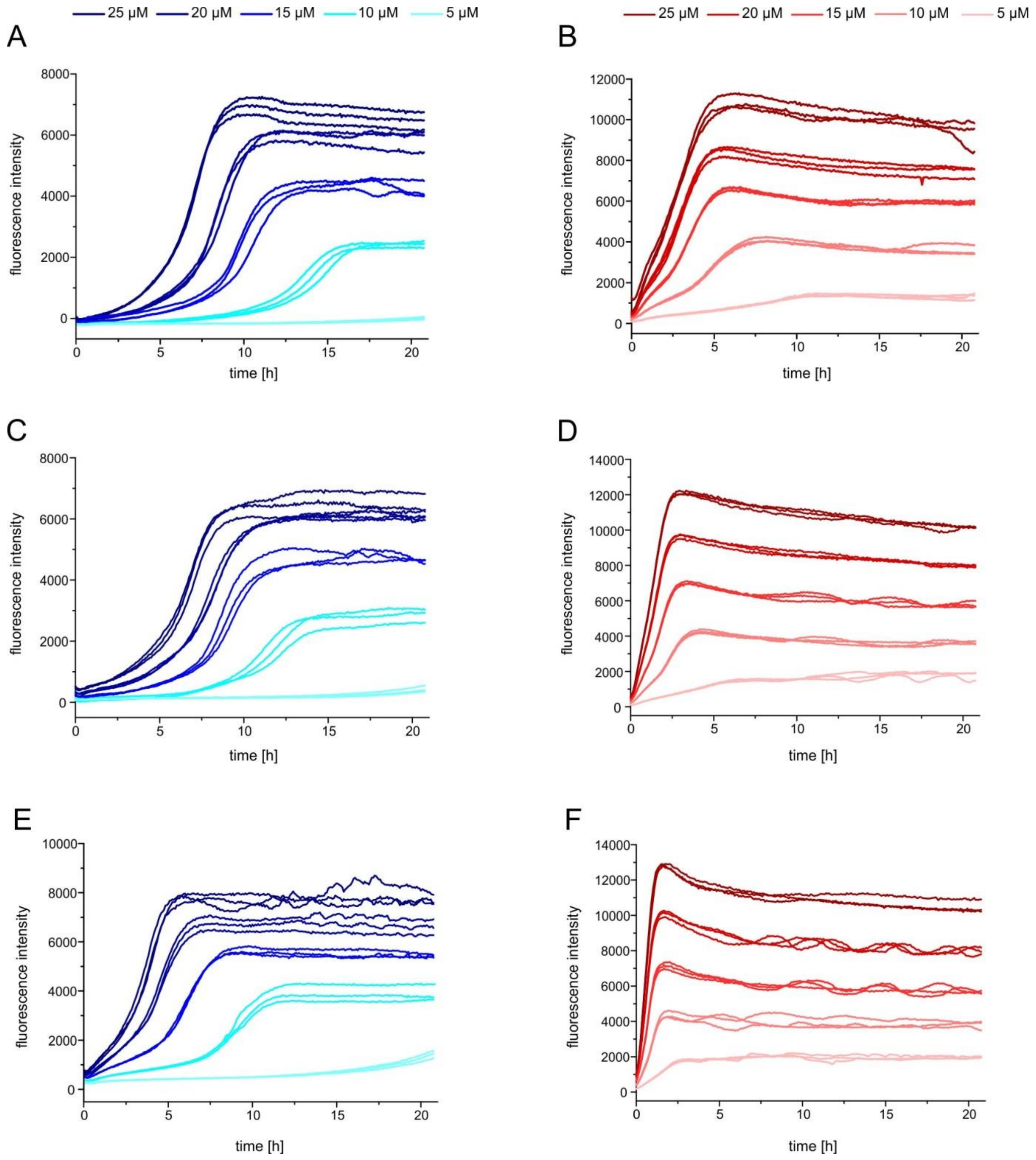

Figure S3. Self- and cross-seeding effects on Aβ(1-42) aggregation kinetics. Raw data of aggregation kinetics of different Aβ(1-42) monomer concentrations seeded with 1, 2 or 5 % Aβ(1-42) (A, C, E) or 1, 2 or 5 % pEAβ(3-42) (B, D, F) fibrils.

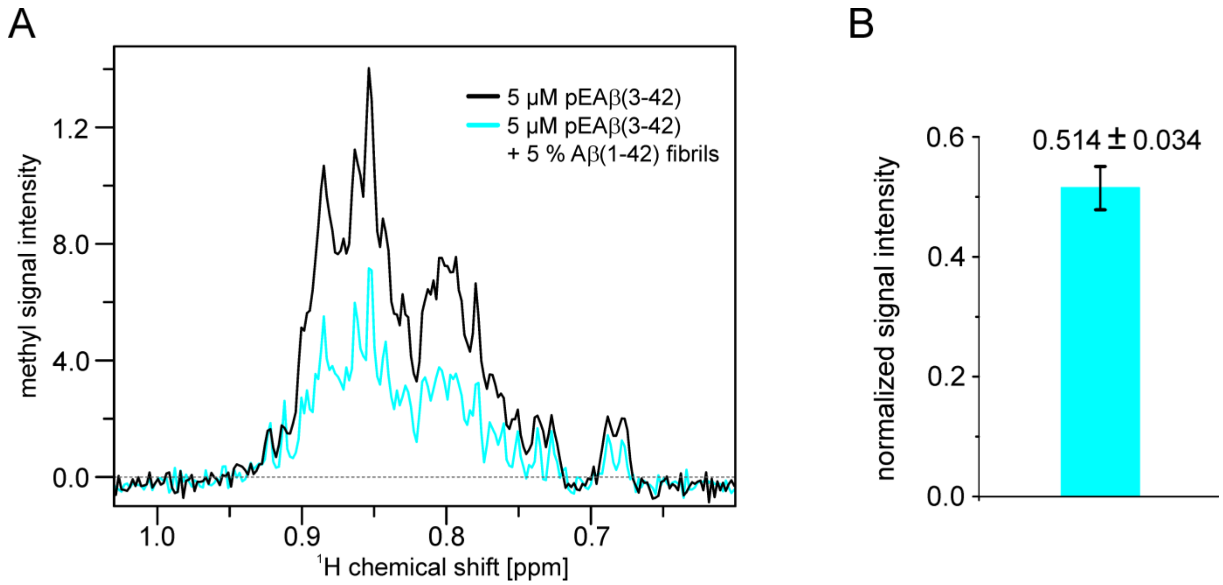

Figure S4. The interaction of Aβ(1-42) fibrils with monomeric pEAβ(3-42) probed by NMR. (A) Methyl signal intensity of 1D-^{1}H-NMR spectroscopy of 5 µM pEAβ(3-42) (black) and 5 µM pEAβ(3-42) to which 5 % Aβ(1-42) fibrils were added (cyan). (B) Normalized methyl signal intensity after addition of the fibrils and standard deviation calculated from the individual peak heights of the obtained spectra.

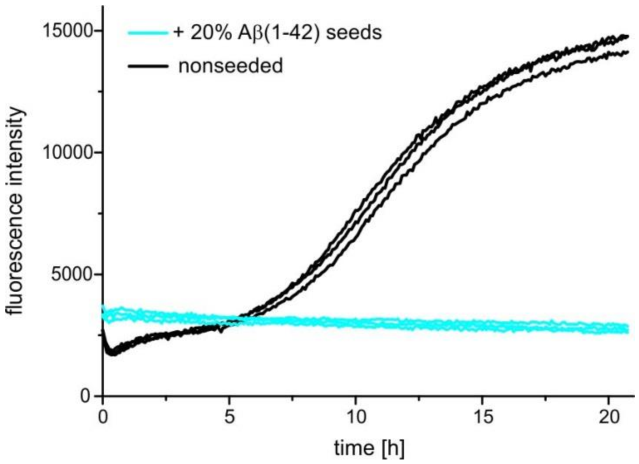

Figure S5. Raw data of aggregation kinetics of an equimolar mixture of 4 µM Aβ(1-42) and pEAβ(3-42) seeded with 20 % Aβ(1-42) fibrils.

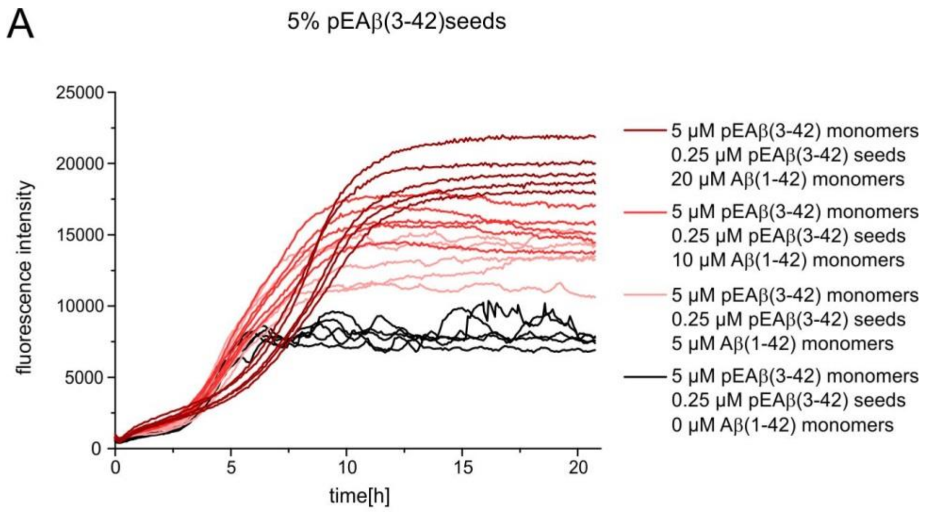

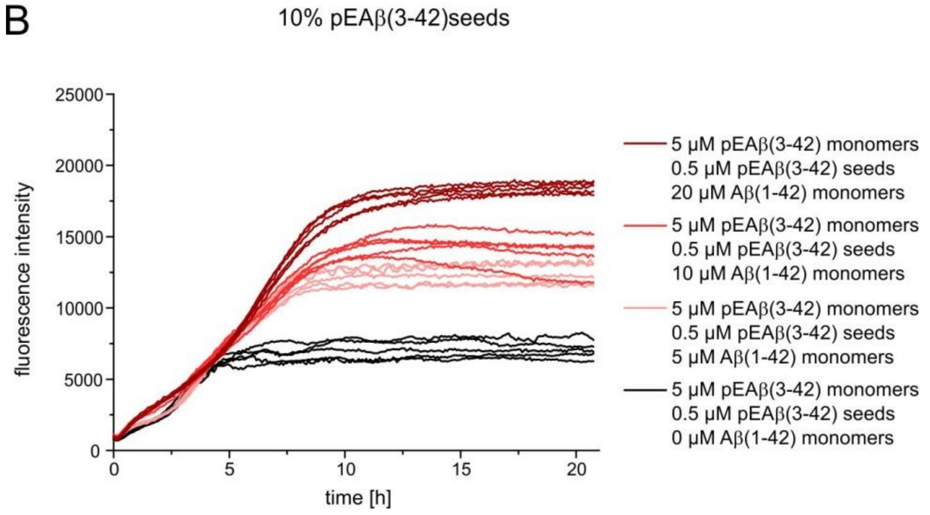

Figure S6. Influence of Aβ(1-42) monomers on pEAβ(3-42) aggregation kinetics. Aggregation kinetics of 5 µM pEAβ(3-42) monomers with varying concentration of Aβ(1-42) monomers and seeded with 5 % (A) or 10 % (B) pEAβ(3-42) fibrils.

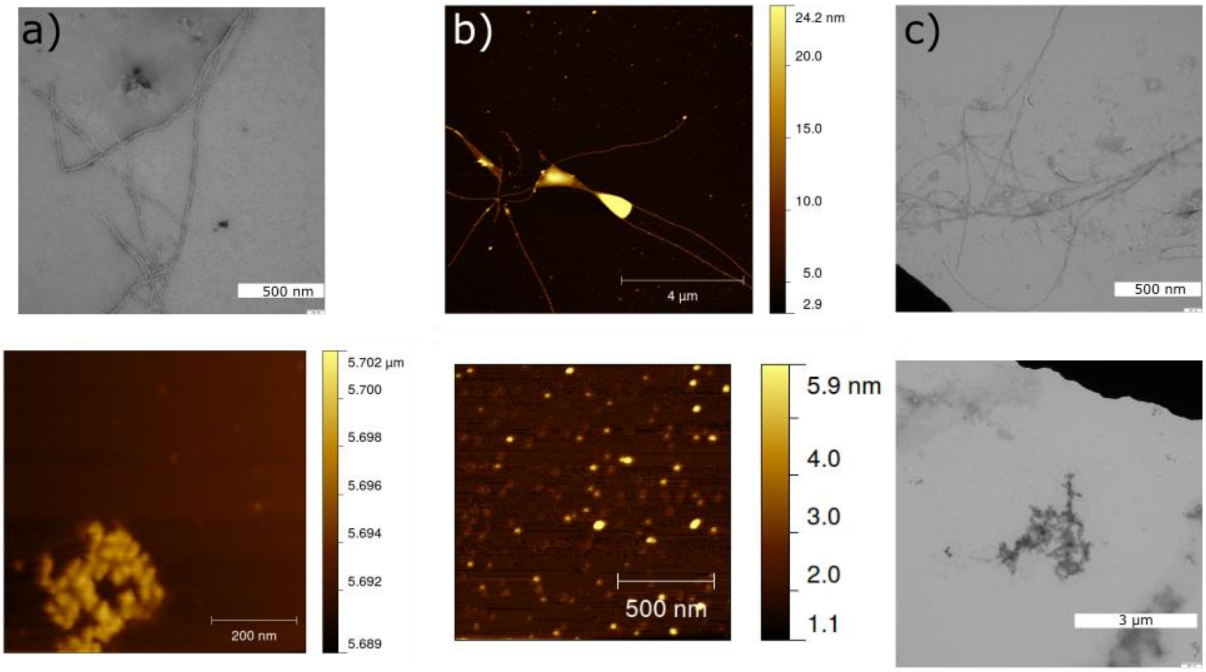

Figure S7. Atomic force microscopy and transmission electron microscopy images of (A) 5 µM Aβ(1-42) and 5 µM pEAβ(3-42), (B) 5 µM Aβ(1-42) and 5 µM pEAβ(3-42) with 5% Aβ(1-42) seeds and (C) 5 µM Aβ(1-42) and 5 µM pEAβ(3-42) with 5% pEAβ(3-42) seeds.

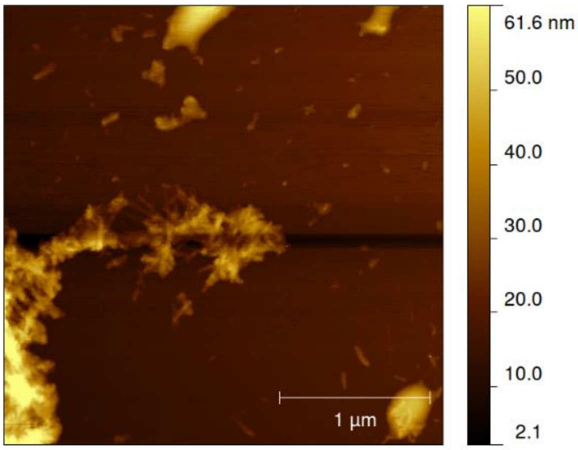

Figure S8. Atomic force microscopy image of 10 µM pEAβ(3-42) with 5% Aβ(1-42) seeds.

(1) Meisl, G.; Kirkegaard, J. B.; Arosio, P.; Michaels, T. C.; Vendruscolo, M.; Dobson, C. M.; Linse, S.; Knowles, T. P. *Nat Protoc* **2016**, *11*, 252.
